# Myostatin blockade with a fully human monoclonal antibody induces muscle hypertrophy and reverses muscle atrophy in young and aged mice

**DOI:** 10.1186/s13395-015-0060-8

**Published:** 2015-10-09

**Authors:** Esther Latres, Jeffrey Pangilinan, Lawrence Miloscio, Roy Bauerlein, Erqian Na, Terra B. Potocky, Ying Huang, Mark Eckersdorff, Ashique Rafique, Jason Mastaitis, Calvin Lin, Andrew J. Murphy, George D. Yancopoulos, Jesper Gromada, Trevor Stitt

**Affiliations:** Regeneron Pharmaceuticals, Inc., 777 Old Saw Mill River Road, Tarrytown, NY 10591 USA

**Keywords:** Myostatin, Hypertrophy, Atrophy, Skeletal muscle, Monoclonal antibody

## Abstract

**Background:**

Loss of skeletal muscle mass and function in humans is associated with significant morbidity and mortality. The role of myostatin as a key negative regulator of skeletal muscle mass and function has supported the concept that inactivation of myostatin could be a useful approach for treating muscle wasting diseases.

**Methods:**

We generated a myostatin monoclonal blocking antibody (REGN1033) and characterized its effects in vitro using surface plasmon resonance biacore and cell-based Smad2/3 signaling assays. REGN1033 was tested in mice for the ability to induce skeletal muscle hypertrophy and prevent atrophy induced by immobilization, hindlimb suspension, or dexamethasone. The effect of REGN1033 on exercise training was tested in aged mice. Messenger RNA sequencing, immunohistochemistry, and ex vivo force measurements were performed on skeletal muscle samples from REGN1033-treated mice.

**Results:**

The human monoclonal antibody REGN1033 is a specific and potent myostatin antagonist. Chronic treatment of mice with REGN1033 increased muscle fiber size, muscle mass, and force production. REGN1033 prevented the loss of muscle mass induced by immobilization, glucocorticoid treatment, or hindlimb unweighting and increased the gain of muscle mass during recovery from pre-existing atrophy. In aged mice, REGN1033 increased muscle mass and strength and improved physical performance during treadmill exercise.

**Conclusions:**

We show that specific myostatin antagonism with the human antibody REGN1033 enhanced muscle mass and function in young and aged mice and had beneficial effects in models of skeletal muscle atrophy.

## Background

Diminished muscle function caused by decreased muscle mass and strength is a key feature of several diseases and disabling conditions and is associated with injury, surgery, frailty, metabolic disorders, and catabolic illnesses such as cancer, heart failure, and chronic lung disease [1, 2]. In the elderly, recovery from orthopedic interventions after a fragility hip fracture for example is significantly impacted by the occurrence of rapid muscle wasting and development of weakness [3]. Therefore, it is essential to develop therapies that increase muscle strength, improve physical performance, and for certain indications, increase survival.

Myostatin plays a central role in the development and maintenance of skeletal muscle, acting as a negative regulator of muscle mass [4, 5]. It is a secreted ligand belonging to the transforming growth factor-β superfamily of growth and differentiation factors and is unique among this family in its specific skeletal muscle expression [4]. Inactivating mutations of the myostatin gene have been described in cattle, sheep, dogs, and humans and result in a profound increase in skeletal muscle mass, without obvious negative effects [6–10]. Targeted deletion of the myostatin gene in mice (*Mstn*^*−/−*^) reproduces the hypermuscular phenotype and results mainly from muscle fiber hyperplasia and also from hypertrophy [4]. *Mstn*^*−/−*^ mice also display significant metabolic improvements including reduced adiposity, increased insulin sensitivity, and resistance to obesity [11–13].

Myostatin is synthesized as a precursor protein, and following processing, mature myostatin is released as a 24-kDa covalent homodimer with its propeptide remaining non-covalently bound, forming an inactive latent complex [5]. Unprocessed precursor and latent complex circulate in the serum [4]. Active myostatin can be released from latent complex by subsequent propeptide cleavage. In serum, myostatin is found in complex with inhibitory proteins, including follistatin, follistatin-like 3, and growth and differentiation factor-associated serum protein-1 (GASP-1) [14, 15]. Myostatin mediates its biological effects primarily through the activin receptor IIB (ActRIIB), which then recruits activin-like kinase-4 (ALK-4) or ALK-5, leading to phosphorylation and activation of the cytoplasmic receptor-regulated Smad2 and 3, which translocate to the nucleus to induce specific gene changes [4, 16].

In this study, we report on the characterization of REGN1033, a fully human monoclonal antibody that inhibits myostatin with sub-nanomolar affinity and high specificity. We demonstrate the efficacy of REGN1033 in increasing muscle mass, strength, and function in both young and aged mice and in models of muscle atrophy, including prevention of disuse atrophy as well as in recovery from pre-existing atrophy. REGN1033 is currently in phase 2 clinical development.

## Methods

### Antibodies and protein reagents

REGN1033 is a fully human monoclonal antibody specific to myostatin derived by immunizing with the mature human myostatin using Regeneron’s VelocImmune® mice [17, 18] in which the myostatin gene was also homozygously ablated, so as to decrease immunotolerance to this protein. The selected anti-myostatin antibody contains an IgG4 constant region. Soluble human ActRIIB-hFc (ActRIIB-hFc) was produced in Chinese hamster ovary (CHO) cells and contains the extracellular domain (1-133) of the human ActRIIB receptor (*ACVR2B*) made as an in-frame fusion with human IgG1 Fc-domain. A second human monoclonal antibody (REGN647) which was shown to be specific for myostatin in binding and bioassay studies was used for immunoblotting; to visualize myostatin propeptide, an anti-propeptide sheep polyclonal antibody (AF1539; R&D Systems: Minneapolis, MN) was used. Human myostatin, growth and differentiation factor 11 (GDF11), and activin A were purchased from R&D Systems.

### Surface plasmon resonance

The affinities of REGN1033 and ActRIIB-hFc for human myostatin, GDF11, and activin A were measured in surface plasmon resonance biacore experiments performed on a Biacore T200 instrument (GE Healthcare, Pittsburgh, PA) using a dextran-coated (CM5) chip at 25 °C. The running buffer was filtered HEPES-buffered Steinberg’s solution (HBS-T) containing 10 mM HEPES, 150 mM NaCl, 3 mM ethylenediaminetetraacetic acid (EDTA), 0.05 % (*v*/*v*) Surfactant P20, 10 mg/ml carboxymethyldextran sodium salt, and 0.5 mg/ml bovine serum albumin (pH 7.4). A capture sensor surface was prepared by covalently immobilizing goat anti-human Fcγ antibody (Jackson ImmunoResearch, West Grove, PA) to the chip surface using 1 ethyl-3-(3-dimethylaminopropyl) carbodiimide hydrochloride/*N*-hydroxysuccinimide (EDC/NHS) coupling chemistry. Following surface activation, anti-human Fc antibody in 0.1 M acetate buffer (pH 4.5) was injected over the activated chip surface until a resonance unit (RU) signal of about 4000 RU (anti-human Fc antibody) was reached. The activated coupled chip surfaces were then washed and treated with 10 mM glycine-HCl (pH 1.5) to remove uncoupled residual proteins. REGN1033 and ActRIIB-hFc were captured through their Fc regions between 32 and 39 RU by an anti-human Fc antibody immobilized on the sensor chips and were tested for binding to the human myostatin, GDF11, and activin A. Myostatin, GDF11, and activin A proteins were prepared at concentrations between 40 and 0.78 nM and individually injected over captured REGN1033 or ActRIIB-hFc surface. All capture surfaces were regenerated with one 30-s pulse of 10 mM glycine-HCl (pH 1.5). Kinetic parameters were obtained by globally fitting the data to a 1:1 binding model using Biacore T200 Evaluation Software. The equilibrium dissociation constant (*K*_D_) was calculated by dividing the dissociation rate constant (*k*_d_) by the association rate constant (*k*_a_).

### Smad2/3 cell-based activity assay

A cell-based assay for determining Smad2/3 activation was established in A204 cells stably expressing Smad-dependent (CAGA12) luciferase. Ligands were serially diluted and added to 20,000 A204/Smad2/3/Luc cells per well plated in McCoy’s 5A medium supplemented with 10 % fetal calf serum (FCS)/G418 to generate a dose-response curve. Inhibition of ligand-mediated ActRIIB signaling by REGN1033, ActRIIB-hFc, or control antibody was tested by serially diluting the antibodies or soluble receptor and incubating with a constant concentration of 1 nM myostatin, 0.4 nM GDF11, or 0.4 nM activin A and cells for 6 h at 37 °C. Luminescence was measured using One Glo (Promega, Madison, WI).

### Human myoblast treatment and immunoblot analysis

Human skeletal myoblasts (Lonza Group Ltd, Anaheim, CA) were cultured in complete growth media (Lonza). Cells (6 × 10^5^ per well in a six-well plate) were cultured for 24 h, and serum starved for 4 h before treated with myostatin, GDF-11, or activin A at 4 nM in presence of 40 nM of REGN1033 or ActRIIB-hFc for 30 min. Cells were lysed in NP-40 buffer containing 1 % NP-40, 100 mM KCL, 20 mM Tris/HCL (pH 7.6), 1 mM EGTA, and 1 mM NaF with various protease and phosphatase inhibitors and cleared by centrifugation. Soluble fractions were separated in 4–20 % Invitrogen Precast Tris-Glycine gels using SDS-PAGE followed by transfer to polyvinylidene fluoride (PVDF) membranes. Total levels of Smad2/3 and phosphorylated Smad2 were determined with rabbit anti-Smad2/3 and phospho-Smad2 (Ser465/467) antibodies (Cell Signaling Technology, Danvers, MA).

### Immunoblot analysis of mouse serum following REGN1033 administration

Serum was collected following 4-week treatment of male CB17-severe combined immunodeficiency (SCID) mice with REGN1033 or control antibody (10 mg/kg by subcutaneous (*s.c*.) dosing; twice in the first week and once a week for three weeks). Serum (60 μl per mouse) was diluted tenfold in phosphate-buffered saline (PBS) containing 0.2 % Nonidet P-40 and 1X protease and phosphatase inhibitors. Immune complexes were captured with Protein A-Sepharose beads and eluted with non-reducing SDS-PAGE buffer. The equivalent of 2.4 μl of serum was then run on a 4–20 % gradient Tris-glycine gel and immunoblotted with a monoclonal antibody against mature myostatin, produced at Regeneron, or with a myostatin propeptide polyclonal antibody. CHO cells stably expressing furin protease were transiently transfected with an expression plasmid encoding human myostatin precursor, and conditioned media was collected after three days; 5 μl was loaded onto the gel to serve as a positive control. To demonstrate myostatin antibody specificity, 2 μl of serum from wild-type and *Mstn*^−/−^ mice was probed by immunoblot.

### Animal studies

Mice (Taconic, Hudson, NY) were housed four to five per cage in a controlled environment (12-h light/dark cycle, 23 ± 1 °C, 60–70 % humidity) and fed ad libitum with standard chow (Purina Laboratory Rodent Diet 5001, LabDiet, St. Louis, MO). All animal studies were conducted in accordance with the Regeneron Pharmaceuticals Institutional Animal Care and Use Committee.

#### Muscle hypertrophy studies

The effect of REGN1033 on muscle mass was determined after 4-week treatment of varying doses of REGN1033 (0.1–30 mg/kg) or control antibody (30 mg/kg) in 9-week-old male CB17-SCID mice (*n* = 5/group). Animals were grouped by body weight and dosed via *s.c.* injection twice the first week and once a week for the following 3 weeks. At the end of the fourth week, tibialis anterior (TA) and gastrocnemius (GA) complex muscle groups were harvested and weighed.

#### Ex vivo force measurements

REGN1033 or control antibody (10 mg/kg) was administered to C57BL/6 male mice (*n* = 6/group) twice a week for 3 weeks via *s.c.* injection. At the end of 3 weeks of treatment, ex vivo force measurements of the TA muscle were obtained. Briefly, mice were anesthetized under isoflurane (4.5 %), and the right TA muscle was excised by cutting the femur just proximal to the femoral head above the knee and the tibia and fibula proximal to the ankle. The muscle was then placed in an oxygenated bath containing Krebs solution with 10 mM glucose at 27 °C. The femoral head was secured to a stanchion while the distal tendon was tied to the arm of a 305C Muscle Lever System (Aurora Scientific, Aurora, ON, Canada). Optimal length was achieved by increasing the length of the muscle by small increments followed by a single 1-Hz stimulation until a maximum twitch force was achieved. Maximal isometric tetanic force was then determined by stimulating each muscle at 10-Hz intervals (from 40 to 100 Hz) with 90-s rest periods prior to each stimulation.

#### Casting immobilization

Two groups of 12-week-old C57BL/6 male mice (*n* = 5/group) were anesthetized, and the right ankle joint was immobilized at a 90° angle with casting material for 14 days. During immobilization, mice were injected subcutaneously twice a week with 30 mg/kg of REGN1033 or control antibody. GA muscle weights were compared to a separate, age-matched, non-immobilized group treated with control antibody (30 mg/kg).

#### Dexamethasone-induced atrophy

Dexamethasone was administered at a rate of 23.0 μg/day by micro-osmotic pump (DURECT Corporation, Cupertino, CA) for 2 weeks in 11-week-old C57BL/6 male mice (*n* = 5/group). During dexamethasone treatment, animals were given either 10 mg/kg of REGN1033 or isotype control antibody by *s.c.* injection twice a week. A separate group, implanted with osmotic pumps delivering saline and given 10 mg/kg of control antibody, served as a negative control. At the end of 2 weeks, TA and GA muscles were collected and weighed.

#### Hindlimb suspension

Prevention of hindlimb suspension (HLS)-induced atrophy was assessed in 10-week-old C57BL/6 male mice (*n* = 8/group). Sixteen mice were tail-suspended for 7 days to prevent weight bearing by the hind limbs; forelimbs were unaffected. Mice were housed in special cages (Techshot Inc., Greenville, IN) with free access to food and water. Another group of mice was left unperturbed to serve as negative controls. Animals were treated with 10 mg/kg of REGN1033 or control antibody by *s.c.* injection 2 days prior to HLS, on the day of HLS, and 4 days into HLS. At the end of 7 days, muscles were collected, weighed, and stored for further analysis. Similarly, the effect of REGN1033 during recovery from 7 days of HLS-induced atrophy was examined in 11-month-old C57BL/6 male mice (*n* = 6/group). A total of eighteen mice were suspended for 7 days, and an unperturbed group was included in the study as a negative control. On day 7 of HLS, the animals were released from suspension and randomized into three groups based on body weight loss. TA and GA muscles were collected and weighed from one HLS group and the unperturbed control group. The remaining HLS animals were allowed to recover for 1 week, receiving either REGN1033 or control antibody at 10 mg/kg twice a week during recovery, at which time TA and GA muscles were collected and weighed.

#### Running endurance in aged mice

Male C57BL/6 at 19 months of age were randomized into four groups (*n* = 6-8/group): a sedentary or exercise group receiving either REGN1033 or control antibody. All mice were dosed via *s.c.* injection twice per week for 3 weeks at 10 mg/kg. During treatment, mice in the exercise group were placed on an exercise regimen involving one training session a day, consisting of 20 min on an Exer 6 M treadmill (Columbus Instruments, Columbus, OH) at 10 m/min with a 5° incline, 5 days a week for three consecutive weeks. At the end of 3 weeks of treatment, endurance was measured in all four groups using a treadmill exhaustion test. Briefly, mice ran on a treadmill at 10 m/min with a 5° incline for 4 min, and the speed was increased by 2 m/min every subsequent 4 min until 16 m/min was reached. At 30 min, the speed was increased to 18 m/min and this speed was maintained until the mice reached exhaustion. Exhaustion was defined as the inability of the mouse to remain on the treadmill despite mechanical prodding and an electrical shock stimulus.

### Histology

Ten-week-old male SCID mice were treated with *s.c.* injection of REGN1033 or control antibody for 4 weeks (10 mg/kg; *n* = 6/group). TA muscles were collected and sectioned, and muscle fibers were stained for laminin with polyclonal rabbit anti-LAMA1 antibody (Sigma-Aldrich, St. Louis, MO). Fiber cross-sectional area was measured using MetaMorph software (Molecular Devices, Sunnyvale, CA). Fiber types were measured in sections of GA muscle that were dried and washed with PBS, quenched in methanol and 1 % H_2_O_2_ solution, blocked with 4 % goat serum and 1 % BSA, and incubated with the following primary antibodies overnight at 4 °C; (1) laminin (1:2000; Sigma), (2) myosin heavy chain slow (1:400; Novocastra, Leica Microsystems Inc., Buffalo Grove, IL), (3) myosin heavy chain 2A (1:500; DSHB, Iowa City, IA), and (4) myosin heavy chain 2B (1:500; DSHB, Iowa City, IA). After a PBS wash, the slides are incubated with the following secondary antibodies from Vector Laboratories (Burlingame, CA); (1) biotinylated goat α-rabbit IgG (1:1200), (2) biotinylated goat α-mouse IgG (1:1000), and (3) biotinylated goat α-mouse IgM (1:1000) for 1 h at 21 °C followed by detection with the ABC kit (1:500; Vector Laboratories). After diaminobenzidine (DAB)/peroxidase brown visualized reaction (0.4 mg/ml DAB, 0.0003 % H_2_O_2_), slides were scanned by the Aperio Scanscope AT. Images were analyzed by HALO software (Indica Labs, Corrales, NM).

### RNA sequencing

Messenger RNA was prepared, sequenced, and analyzed as previously described [19].

### Statistical analysis

Data are presented as mean ± standard error, and values of *P* < 0.05 were considered statistically significant. Statistical significance was measured through unpaired, two-tailed Student’s *t* test for comparisons between two groups, one-way or two-way ANOVA with Tukey’s post hoc analysis for studies with group of three or more or two-way repeated measures ANOVA with Bonferroni’s post hoc analysis for studies where time was a factor using Prism software (GraphPad Software).

## Results

### In vitro characterization of REGN1033 anti-myostatin antibody

REGN1033 was selected to have high affinity to myostatin (*K*_*D*_ = 24 pM) (Table 1). This applies to mouse, rat, monkey, and human since myostatin is 100 % conserved across these species. No binding was detected between REGN1033 and human GDF11 or activin A. ActRIIB-hFc exhibited high affinity binding to GDF11 and activin A with *K*_D_ values comparable to those measured for myostatin (*K*_D_ = 14-87 pM) (Table 1).

**Table 1:** Kinetic binding parameters for REGN1033 and ActRIIB-hFc binding to human myostatin, GDF11 and activin A determined by SPR-Biacore

Myostatin inhibitors	Ligand	Kinetic binding parameters			
		*k*_a_ (M^−1^s^−1^)	*k*_d_ (s^−1^)	*K*_D_ (pM)	T_1/2_ (min)
REGN1033	GDF8	1.23 × 10^6^	2.93 × 10^−5^	23.9	394
	GDF11	NB	NB	NB	NB
	Activin A	NB	NB	NB	NB
ActRIIB-hFc	GDF8	5.98 × 10^6^	1.00 × 10^−4^	16.8	115
	GDF11	7.38 × 10^6^	1.03 × 10^−4^	14.0	112
	Activin A	9.17 × 10^5^	7.94 × 10^−5^	86.6	146

*NB* no detectable binding

**Fig. 1:**
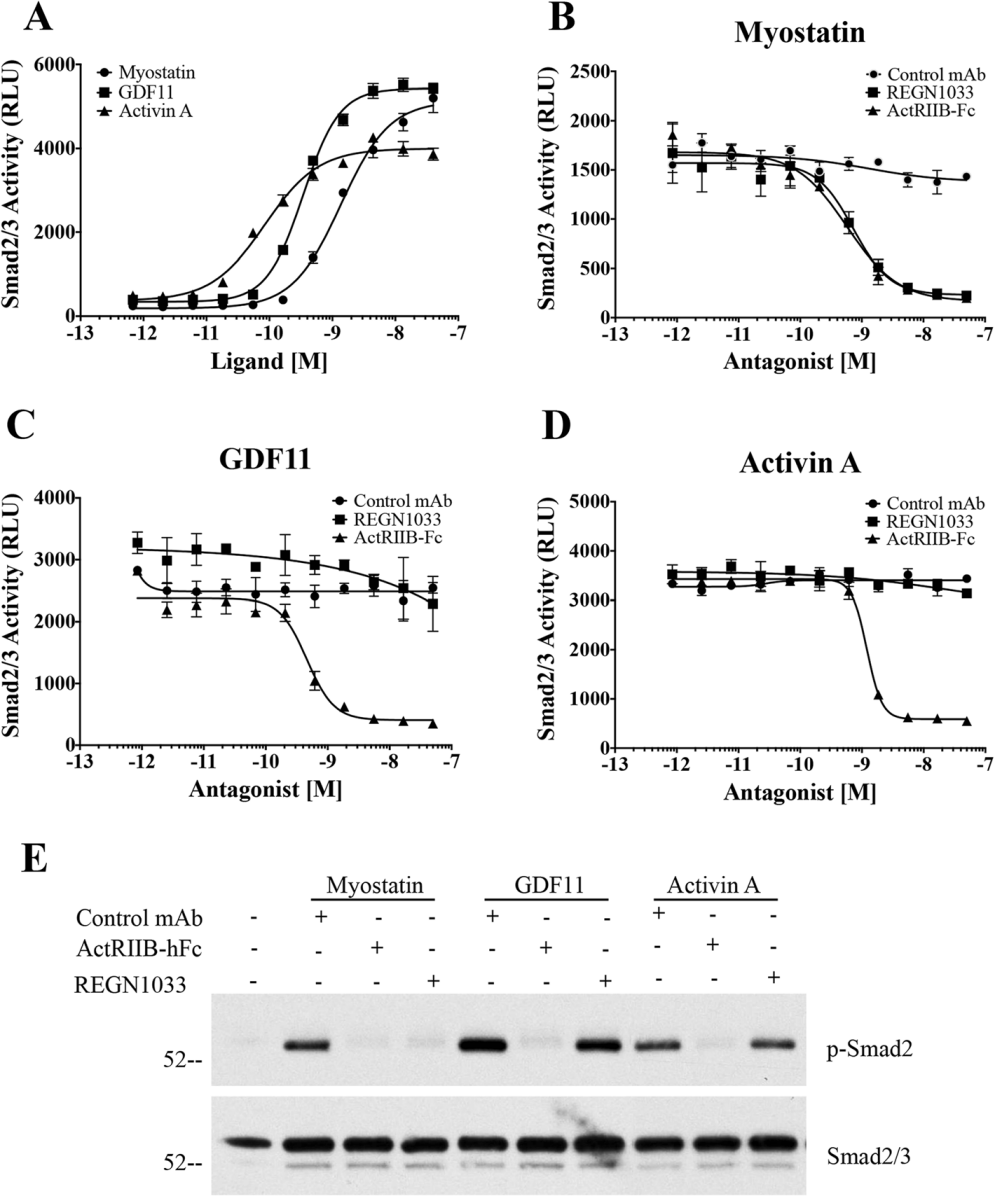
Effects of myostatin, GDF11, and activin A on Smad2/3 activity. **a** Smad2/3 activity was recorded as relative luminescence units (RLU) from A204/Smad2/3/Luc reporter cells in the presence of increasing concentrations of myostatin, GDF11, or activin A. Smad2/3 activity in the presence of constant myostatin (**b**), GDF11 (**c**), or activin A (**d**) concentrations and increasing concentrations of control antibody (*circles*), REGN1033 (*squares*), and ActR2B-hFc (*triangles*). **e** Phospho-Smad2 (p-Smad2) and total Smad2/3 western blot from human skeletal myoblasts in the absence and presence of myostatin, GDF11, or activin A and either control antibody (Control mAb), ActRIIB-hFc, or REGN1033

In the A204 cell-based bioassay, myostatin, GDF11, or activin A stimulated Smad2/3 signaling with 50 % of the maximum activity (EC_50_) at 1.37 nM for myostatin, 0.34 nM for GDF11, and 0.09 nM for activin A (Fig. 1a). REGN1033 effectively blocked receptor activation by a constant concentration of 1 nM myostatin (IC_50_ = 0.73 nM) but not by constant concentrations of 0.4 nM GDF11 or activin A (Fig. 1b–d). ActRIIB-hFc blocked receptor activation caused by all three ligands (myostatin; IC_50_ = 0.57 nM, GDF11; IC_50_ = 0.46 nM, and activin A; IC_50_ = 1.2 nM) (Fig. 1b–d).

Consistent with the above data, we found that myostatin, GDF11, and activin A increased phosphorylated Smad2 (p-Smad2) levels in lysates from human primary skeletal myoblast (Fig. 1e). REGN1033 selectively reduced p-Smad2 levels induced by myostatin, whereas ActRIIB-hFc blocked Smad2 activation by all three ligands (Fig. 1e). These data show that REGN1033 binds myostatin with sub-nanomolar affinity and high specificity and blocks myostatin-induced Smad2/3 signaling.

### REGN1033 increases muscle hypertrophy and force in mice

**Fig. 2:**
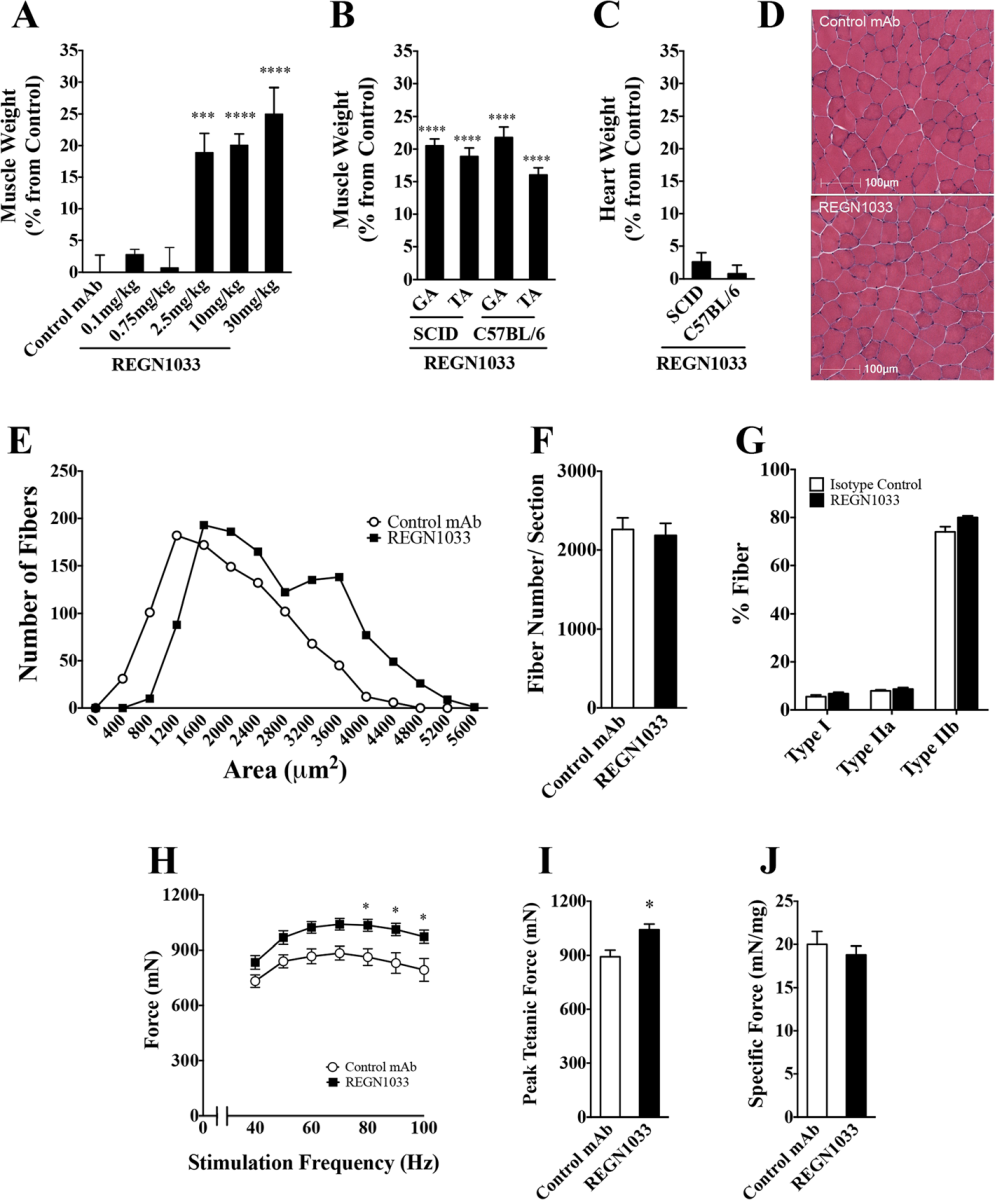
Effects of REGN1033 on muscle mass, muscle fiber area, and number and force. **a** Gastrocnemius anterior (GA) muscle weight after 4 weeks of treatment with REGN1033 at 0.1, 0.75, 2.5, 10, or 30 mg/kg in CB17-SCID mice. Irrelevant isotype antibody was used as control. Muscle weights were normalized by starting body weights and expressed as percent changes from isotype control. **b** GA and tibialis anterior (TA) muscle weights normalized by starting body weight and expressed as percent changes from isotype control in CB17-SCID and C57BL/6 mice dosed at 10 mg/kg for 3–4 weeks. **c** Heart weights normalized by starting body weight and expressed as percent changes from isotype control in CB17-SCID mice dosed at 10 mg/kg for 4 weeks. **d** H&E staining of TA muscle from isotype control antibody and REGN1033-treated CB17-SCID mice for 4 weeks. Muscle fiber size distribution (**e**), fiber count (**f**), and fiber type (**g**) of GA muscle from CB17-SCID mice after 4-week treatment with 10 mg/kg of control antibody or REGN1033. Muscle fiber type and number are representatives of five independent studies. **h** Isometric force as a function of stimulation frequency of TA muscle at the end of 3 weeks of treatment with isotype control antibody or REGN1033. Peak tetanic force (**i**) and specific force (**j**) from the experiment shown in **h**. All groups had 5–10 animals. Data are means ± SEM. *P < 0.05, ***P < 0.001, ****P < 0.0001 isotype vs. control

The effect of REGN1033 on skeletal muscle mass was determined in CB17-SCID male mice following 28 days of treatment with REGN1033 (0.1, 0.75, 2.5, 10, or 30 mg/kg) or control antibody (10 mg/kg). Figure 2a shows GA muscle weights normalized to body weights at the start of the experiment and depicted as the percent change compared to control. Body weights increased 3–7 % in the REGN1033-treated animals relative to the control group (data not shown). REGN1033 elicited significant increase in muscle weights at 2.5 mg/kg and higher doses, producing GA muscle weight increases of 19–25 % (Fig. 2a). Aggregate data from 17 studies revealed that treatment with 10 mg/kg of REGN1033 for 3–4 weeks increased GA muscle weight by 20.8 ± 0.6 % (*P* < 0.0001). REGN1033 produced a similar increase in TA muscle weight from CB17-SCID mice (18.9 ± 1.3 %; *P* < 0.0001) (Fig. 2b). Comparable increases in GA and TA muscles was observed in REGN1033 treated C57BL/6 mice (Fig. 2b). Heart weight did not significantly changed with REGN1033 treatment in either strain of mice (Fig. 2c).

To assess the effects of REGN1033 on muscle fiber area and number, CB17-SCID mice were administered REGN1033 or control antibody (10 mg/kg) for 4 weeks and the TA muscle was removed for histological analysis (Fig. 2d). Comparison of the frequency distribution of muscle fiber cross-sectional area showed a shift towards higher fiber diameter (Fig. 2e). On average, data from five independent studies showed that REGN1033 increased muscle fiber area by 15.4 ± 3.1 % (*P* < 0.001; *n* = 26) relative to control antibody-treated animals. Muscle fiber number and type did not change (Fig. 2f, g).

In a separate study, C57BL/6 mice were treated with REGN1033 or control antibody (10 mg/kg) for 3 weeks and TA muscle was isolated for ex vivo isometric force measurements. Figure 2h shows that REGN1033 increased isometric force production at all stimulation frequencies. Maximal tetanic force was increased by 16.7 % (*P* < 0.05, *n* = 6) compared to force measured in muscle from control antibody-treated mice (Fig. 2i). Specific force generation remained unchanged between muscle from control antibody and REGN1033-treated mice (Fig. 2j). These data show that REGN1033-induced muscle hypertrophy translates into an increase in muscle fiber cross-sectional area and force.

### REGN1033 binds mature myostatin, latent complex, and pro-myostatin

**Fig. 3:**
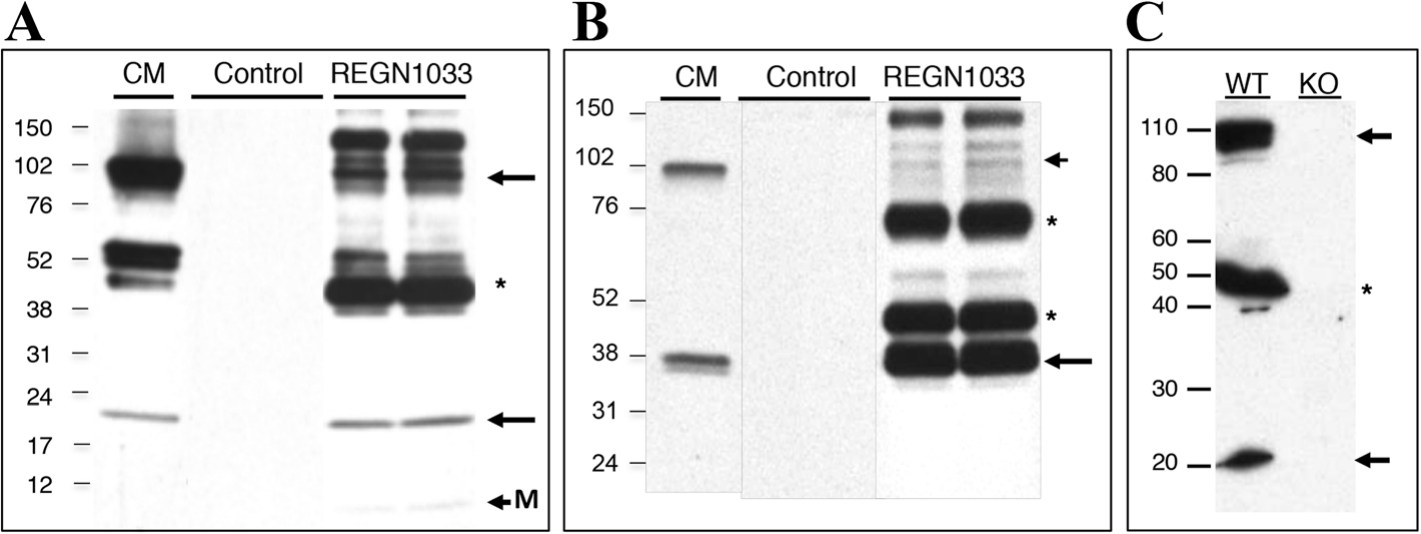
Myostatin, latent complex, and precursor forms bound by REGN1033 in vivo. **a** Antibody complexes present in serum samples from C17-SCID mice that had received multiple REGN1033 or isotype-matched control antibody injections over 28 days were collected by immunoprecipitation, separated by non-reducing SDS-PAGE, and immunoblotted with a myostatin antibody. The *first lane* contains conditioned medium from CHO cells transiently transfected with myostatin precursor expression vector to serve as a positive control; two independent serum immunoprecipitations from control or REGN1033-treated mice are shown. *Arrows* indicate mature myostatin homodimer, unprocessed precursor, and myostatin monomer (*M*). *Asterisk* indicates myostatin form believed to be partially processed precursor. All samples were resolved on the same gel and appropriate lanes are juxtaposed for clarity. **b** Duplicate samples as described in **a** were probed with a myostatin propeptide antibody. *Arrow* indicates cleaved prodomain which co-precipitates in complex with mature myostatin, *arrowhead* indicates unprocessed precursor, and *asterisks* indicate partially processed precursor forms. **c** Sera from WT and *Mstn* ^*−/−*^ mice was probed with the myostatin antibody to demonstrate specificity. *Arrows* indicate myostatin precursor and mature myostatin homodimer, and the *asterisk* indicates partially processed precursor, all of which are absent in the *Mstn* ^*−/−*^ sample

Myostatin in circulation exists primarily as a latent complex consisting of the mature homodimer bound non-covalently by its propeptide; unprocessed dimeric pro-myostatin and an apparently partially processed form are also seen [5]. To see if REGN1033 forms stable complexes with myostatin in vivo, we performed pull-down assays. Immunoblot analysis of Protein A-Sepharose precipitates of serum samples from REGN1033 or control antibody-treated mice (10 mg/kg; 28 days) with an antibody against mature myostatin demonstrates that REGN1033 binds (1) mature myostatin (~22 kDa), (2) unprocessed precursor (~100 kDa), and (3) partially processed latent complex (~50 kDa); a faint band migrating at the expected molecular weight of myostatin monomer (~12 kDa) was also detected (Fig. 3a). None of these species were present in serum samples from mice-injected with control antibody. These myostatin forms are also present in the positive control lane obtained from myostatin-transfected CHO cells (Fig. 3a). A separate propeptide-specific immunoblot of the same samples demonstrates the ability of REGN1033 to bind myostatin when it is bound in a latent complex with its prodomain, i.e., the prodomain is specifically co-immunoprecipitated along with mature myostatin (Fig. 3b). To demonstrate specificity of the myostatin immunoblotting antibody, the antibody was used to probe serum from wild-type (WT) and *Mstn*^*−/−*^ mice (KO) (Fig. 3c); myostatin precursor, mature homodimer, and partially processed forms are seen in the WT sample and are absent from the myostatin KO sample.

### Gene expression analysis in muscle from REGN1033-treated mice

**Fig. 4:**
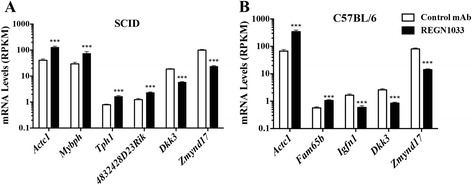
Gene expression in GA muscle from REGN1033 treated mice. Gene changes (twofold, *p* < 0.01) in GA muscle from CB17-SCID (**a**) and C57BL/6 (**b**) mice dosed at 10 mg/kg for 3 to 4 weeks. Results are expressed in reads per kilobase per million (RKPM). Data are means ± SEM of four mice

We analyzed mRNA levels of genes expressed in GA muscle from CB17-SCID and C57BL/6 mice treated with REGN1033 or control antibody (10 mg/kg) for 3 to 4 weeks. Surprisingly, a total of only eight genes showed a change in relative mRNA levels of ≥twofold (*P* < 0.01) in response to REGN1033 treatment (Fig. 4). mRNA level of one gene was upregulated (*Actc1*), whereas the levels of two genes were reduced (*Dkk3* and *Zmynd17*) in both strains of mice. Expression levels of three genes (*Mybph*, *Tph1*, and *4832428D23Rik*) were specifically upregulated in CB17-SCID mice (Fig. 4a). The mRNA level of *Fam65b* was increased, whereas expression of *Igfn1* was reduced in C57BL/6 mice (Fig. 4b).

### REGN1033 prevents muscle atrophy and spares muscle force

**Fig. 5:**
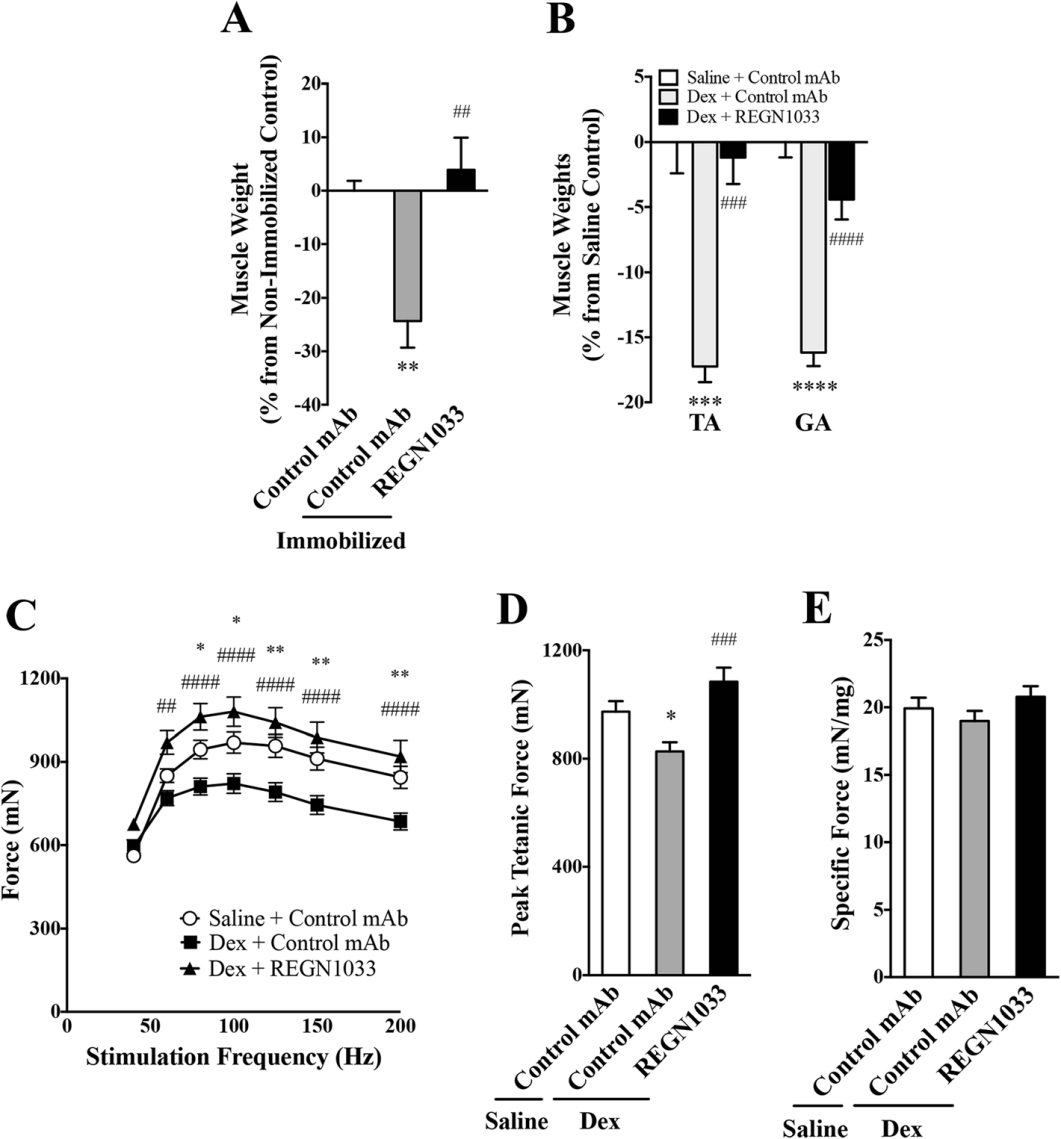
REGN1033 prevents muscle atrophy by immobilization and dexamethasone treatment. **a** GA muscle weights from mice in which the right hindlimbs were immobilized for 14 days. REGN1033 or isotype control antibody was dosed during the 14-day immobilization period. Results are expressed as the percent change compared to muscle weights of non-immobilized limbs. Statistical significance vs. non-immobilized control is indicated. **b** TA and GA muscle weights from C57BL/6 mice implanted with osmotic pumps delivering saline or dexamethasone (Dex; 23 μg/day) and simultaneously treated with REGN1033 or isotype control antibody (10 mg/kg) for 2 weeks. Results are presented as the percent change compared to muscle weights of the saline pump control group. Statistical significance vs. saline control is indicated as *asterisk*, whereas significance vs. Dex plus isotype control is indicated as *number sign*. **c** Isometric force as a function of stimulation frequency of TA muscle from mice implanted with osmotic pumps delivering saline or dexamethasone (Dex) and treated with REGN1033 or isotype control antibody (10 mg/kg) for 2 weeks. Statistical significance vs. saline control is indicated as *asterisk*, whereas significance vs. Dex plus isotype control is indicated as *number sign*. Similar to **c** but depicting peak tetanic force (**d**) or specific force (**e**). All groups had 5–12 animals. Data are means ± SEM. *P < 0.05, **P < 0.01, ***P < 0.001, ****P < 0.0001 and ^##^ *P* < 0.01, ^###^ *P* < 0.001, ^####^ *P* < 0.0001

To determine if REGN1033 prevents skeletal muscle atrophy, we performed studies in models of atrophy induced by casting immobilization or dexamethasone administration. Hindlimb casting for 2 weeks reduced GA muscle mass by −24.3 ± 4.9 % (*P* < 0.01, *n* = 5) compared to non-immobilized limb receiving control antibody (Fig. 5a). In contrast, immobilized mice treated with REGN1033 showed an increase in GA muscle mass by 3.9 ± 6.0 % (*n* = 5) over GA muscle from non-immobilized control antibody-treated mice. This represents a complete prevention of muscle loss by REGN1033.

The ability of REGN1033 to prevent muscle wasting was also examined in glucocorticoid-induced atrophy (Fig. 5b). In the presence of control antibody, 2-week treatment of C57BL/6 mice with dexamethasone-induced muscle atrophy as measured by the loss of TA (−17.2 ± 1.2 %; *P* < 0.001, *n* = 5) or GA (−16.2 ± 1.0 %; *P* < 0.0001, *n* = 5) muscle mass. In contrast, REGN1033 (10 mg/kg) prevented the loss of muscle mass induced by dexamethasone in both muscle types (Fig. 5b). Importantly, ex vivo force analysis of the TA muscle demonstrated that the atrophy seen was accompanied by loss of isometric force production and that this functional loss was alleviated by REGN1033 (10 mg/kg; Fig. 5c). Maximal tetanic force was reduced by −15.0 % by dexamethasone and prevented in the REGN1033 treatment group (11.8 % increase above saline treated mice) (Fig. 5d). Specific force remained unchanged in muscle from control antibody-treated mice receiving saline or dexamethasone as well as dexamethasone and REGN1033 (Fig. 5e).

### REGN1033 prevents disuse muscle atrophy and improves recovery time in hindlimb suspension

**Fig. 6:**
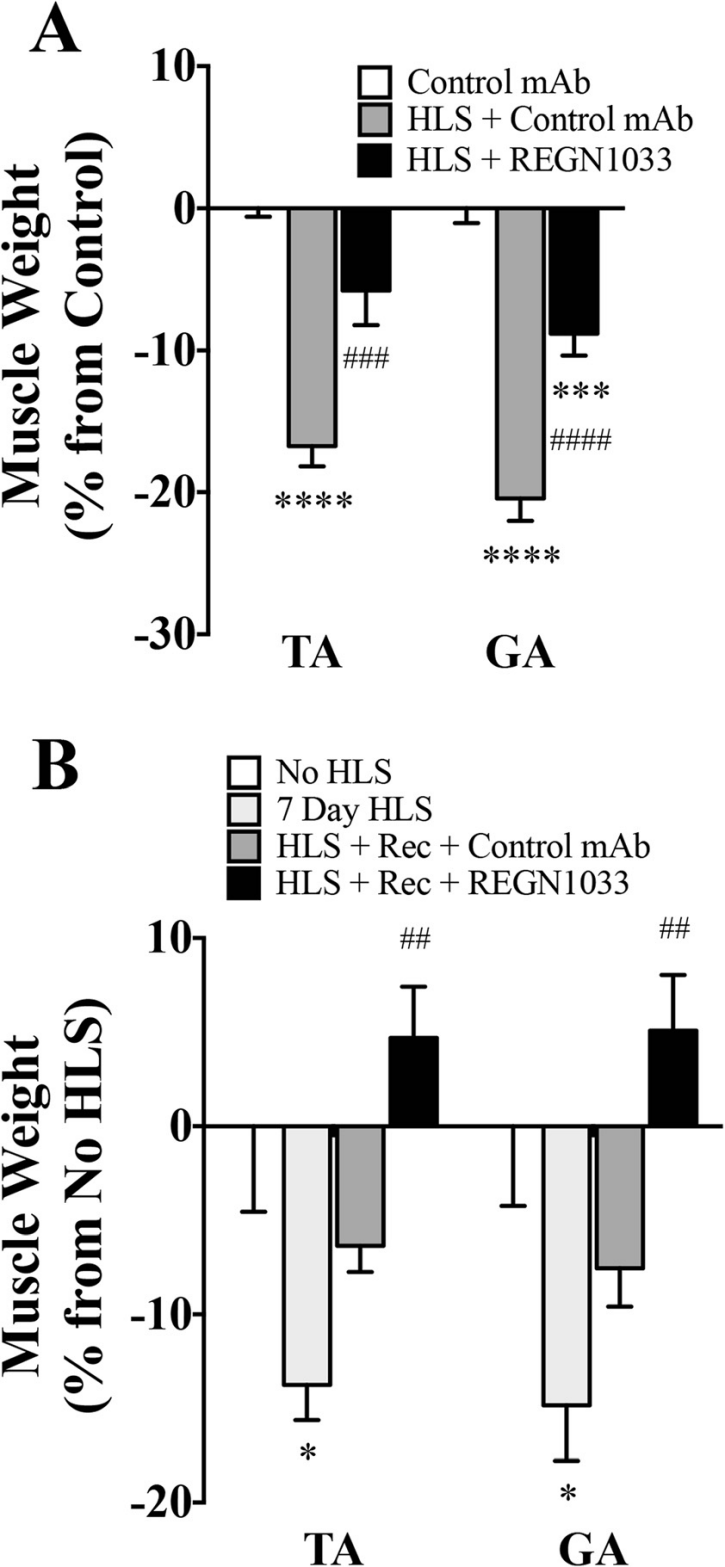
Effects of REGN1033 on muscle atrophy induced by hindlimb unloading. **a** TA and GA muscle mass after treatment with REGN1033 or an isotype control antibody during 7 days of hindlimb unloading (HLS). One group of mice served as control to the HLS group and received injections with the isotype control antibody. Weights were normalized according to starting body weight, and data are expressed as percent change from normalized non-hindlimb suspended control group. Significance was calculated over normalized non-hindlimb suspended isotype control group (asterisk) or over normalized hindlimb suspended isotype control group (number sign). **b** Muscle weights at the end of 7 days of hindlimb unloading (HLS) or 7 days of HLS plus 7 days of recovery (HLS + rec). Groups of mice were dosed with isotype control or REGN1033 (10 mg/kg) during the 7-day recovery period. Changes in muscle weights are depicted as the difference in muscle weights compared to the no-hindlimb-suspended group. Statistical significance was calculated vs. animals that were not-hindlimb-suspended (no HLS) or against animals that were hindlimb-suspended for 7 days (7-day HLS). All groups had four to six animals. Data are means ± SEM. *P < 0.05, **P < 0.01, ****P < 0.0001 and ^##^ *P* < 0.01, ^###^ *P* < 0.001, ^####^ *P* < 0.0001

Next, we determined the ability of REGN1033 to prevent muscle atrophy and improve recovery following an extended period of hindlimb suspension. When control antibody (25 mg/kg) was administered during hindlimb suspension, mice lost −16.7 ± 1.4 % (*P* < 0.0001, *n* = 7-8) of TA muscle mass and −20.4 ± 1.6 % (*P* < 0.0001, *n* = 7-8) of GA muscle mass (Fig. 6a). Treatment with REGN1033 (25 mg/kg) during hindlimb suspension prevented the loss of muscle mass by 65 % in TA and 57 % loss in GA muscle (Fig. 6a).

To test the effects of REGN1033 during the recovery phase from hindlimb suspension, we utilized 1 year-old mice, since they show slower recovery in regaining muscle mass than young mice. Seven days of hindlimb suspension in these old mice decreased muscle mass by −12.7 ± 3.7 % (*P* < 0.05, *n* = 5) in TA and −13.7 ± 4.9 % (*P* < 0.05, *n* = 5) in GA muscle (Fig. 6b). Upon hindlimb suspension cessation, mice that received control antibody during the 7-day recovery phase regained half of their muscle mass (Fig. 6b). In contrast, the presence of REGN1033 (10 mg/kg) during this phase significantly augmented recovery, resulting in muscle weights higher than those of unperturbed mice (Fig. 6b).

### REGN1033 increases muscle mass and strength and running endurance in aged-year-old mice

**Fig. 7:**
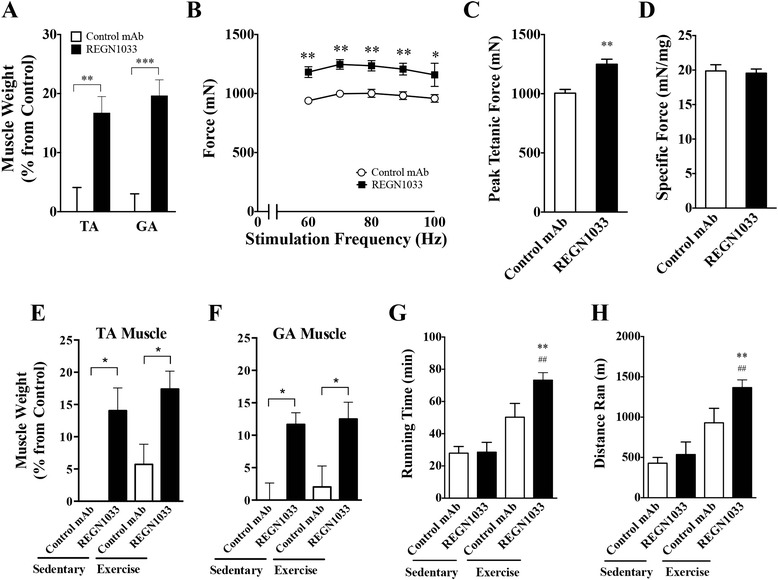
Effects of REGN1033 on muscle mass and exercise performance in aged mice. **a** TA and GA muscle weights of 24-month-old mice receiving REGN1033 or isotype control antibody treatment (10 mg/kg) for 3 weeks. Muscle weights were normalized by starting body weights and expressed as percent changes from isotype control. **b** Isometric force as a function of stimulation frequency of TA muscle at the end of 3 weeks of treatment with isotype control antibody or REGN1033. Same as in **b** but depicting peak tetanic TA muscle force (**c**) or specific force (**d**). TA (**e**) and GA (**f**) muscle weights at the end of 3 weeks of treatment with REGN1033 or isotype control antibody (10 mg/kg) from 19-month-old C57BL/ mice either left sedentary or during a three week exercise regimen. Muscle weights were normalized by starting body weights and expressed as percent changes from isotype control. **g**, **h** Treadmill exhaustion and maximal running capacity tests measured in the mice described in **e**, **f**. All groups had six to eight animals. Data are means ± SEM. *P < 0.05, **P < 0.01, ****P < 0.0001 vs. sedentary isotype control and ^##^ *P* < 0.01 vs. sedentary REGN1033

Decrease in muscle strength and function are considered a consequence of age-related sarcopenia. As inhibition of myostatin with REGN1033 could potentially be useful for counteracting the progressive loss of muscle mass and function in the aging population, we tested the efficacy of REGN1033 in 24 month-old C57BL/6 mice. REGN1033 (10 mg/kg) induced a significant increase in TA and GA muscle mass (Fig. 7a). No change in heart weights was observed (data not shown). Importantly, TA muscles from aged mice treated with REGN1033 showed a significant increase in the maximum force generated (Fig. 7b, c). Specific muscle force remained unchanged between the treatment groups (Fig. 7d). These data show that REGN1033 increases muscle mass and force in aged mice.

We next evaluated the effects of REGN1033 in a model of muscle endurance, measuring muscle mass and exercise performance in aged male C57BL/6 mice at 19 months of age. A running exercise protocol was used during a 3-week period during which mice received 10 mg/kg of REGN1033 or control antibody. Two additional groups of mice receiving REGN1033 or control antibody (10 mg/kg) were used as sedentary controls. Figure 7e shows that exercise training did not increase muscle mass and that REGN1033 induced comparable muscle hypertrophy over controls in both sedentary animals (11.6 % increase over controls) and in exercised animals (11.0 % increase over controls), indicating that exercise alone did not increase muscle mass in aged mice and that exercise is not required for the ability of REGN1033 to induce hypertrophy. In exercised aged mice, it appeared as though REGN1033 increased endurance, as indicated by strong trend toward increased treadmill running time and distance when compared with exercised control mice (Fig. 7f, g). These results suggest that REGN1033 can increase physical performance outcomes above that achieved with exercise training in aged mice.

## Discussion

We report the development and characterization of REGN1033, a fully human monoclonal antibody that has high affinity and specificity to myostatin. In vivo, REGN1033 (1) induced skeletal muscle hypertrophy and increased muscle force in young and aged mice, (2) prevented the loss of skeletal muscle mass in models of atrophy and accelerated the recovery of muscle mass from pre-existing atrophy, and (3) improved physical performance outcomes in combination with treadmill exercise in 2-year-old mice. Our results indicate that specific blockade of myostatin with REGN1033 has the potential to provide a safe and effective therapy for human diseases that are associated with decreased skeletal muscle mass and function in both young and elderly individuals.

It has previously been reported that administration of myostatin-directed monoclonal antibodies in adult mice increases muscle mass and strength and attenuates muscle atrophy [20–25]. It is important to note that these antibodies either blocked additional transforming growth factor beta (TGFβ) family members or their specificity was not reported. While blockade of multiple TGFβ ligands may provide a useful approach to maximize efficacy in muscle wasting conditions, these ligands have a diverse range of target tissues and biological roles, many of which are not fully understood. Here, we report that REGN1033 inhibits myostatin with high affinity and specificity. Indeed, REGN1033 does not bind or block GDF11, which is more than 90 % identical to mature myostatin. The lack of cross-reactivity to GDF11 is important, since data suggest that GDF11 has beneficial effects in aged skeletal muscle and reverses age-related cardiac hypertrophy [26, 27], so that blockade of GDF11 would not be desirable. However, these findings have recently been challenged and suggest that GDF11 increases with age and inhibits muscle regeneration [28]. Few gene changes were observed in muscle from REGN1033-treated mice. This emphasizes the specificity of REGN1033 for myostatin and that a specific gene program is turned on to promote muscle growth. *Zmynd17* is the most downregulated gene in TA muscle from both SCID and C57BL/6 mice. Based on these findings, we generated *Zmynd17*-deficient mice. Comparison between *Zmynd17* deficient and wild-type littermates did not reveal differences in body weight, glucose homeostasis, as well as skeletal muscle mass and function (data not shown). This suggests that down regulation of *Zmynd17* expression is a consequence rather than a cause of muscle growth in response to REGN1033 administration.

REGN1033 displays the ability to recognize and bind multiple circulating forms of myostatin including the latent complex and unprocessed precursor. While release of active myostatin from its inactive latent form is thought to occur through BMP1-type proteolytic cleavage of its propeptide [29], little is known about the in vivo regulation of this activation step or the fraction of free bioactive myostatin present in circulation. Thus, REGN1033 provides an opportunity to limit the signaling potential of latent pools of myostatin, independent of subsequent activation steps.

Administration of REGN1033 antibody at doses as low as 2.5 mg/kg in mice increased muscle mass by 15–25 %, with no significant effect on heart weight. Myostatin is expressed in the adult heart at very low levels [4, 30]. The increase in skeletal muscle mass is due to fiber hypertrophy without fiber hyperplasia or fiber-type switching and results in higher muscle isometric force. The increase in muscle mass is similar to that observed following post-developmental [31] inactivation of myostatin for 3–4 months [32] and slightly less than following follistatin overexpression, which results in inactivation of both myostatin and activin A [33]. Finally, in adult myostatin null mice, the two- to threefold increase in skeletal muscle mass results primarily from fiber hyperplasia and also from a 10–30 % increase in fiber hypertrophy [34–36]. These data suggest that in adult mice, REGN1033 fully blocks myostatin action in vivo and causes a maximal increase in muscle hypertrophy. The data also imply that the primary role of myostatin during development is to regulate satellite cell activation and fusion to myofibers and that this mechanism does not seem to play a significant role for changes in skeletal muscle mass following myostatin inhibition in the adult mouse in physiological settings [31, 37]. In addition to inducing hypertrophy in unperturbed animals, REGN1033 also prevented the loss of skeletal muscle mass in models of muscle atrophy, such as hindlimb suspension, casting immobilization, and dexamethasone treatment. Importantly, REGN1033 also prevented the loss of muscle strength in atrophy settings and accelerated recovery from pre-existing atrophy.

There is a large unmet medical need for treatment options for alleviation of muscle atrophy in elderly patients recovering from orthopedic disuse following joint replacement or repair surgery and in those patients impacted by the frailty and morbidity associated with generalized sarcopenia. We show that antagonism of myostatin with REGN1033 not only increases muscle mass and strength in aged-month-old mice similar to that observed in young mice but also improves exercise endurance as measured by the time and distance that mice ran on a treadmill before exhaustion.

## Conclusions

In summary, our data provides further support for the notion that specific antagonism of myostatin can enhance skeletal muscle mass and function in normal mice and multiple settings of atrophy. Of interest, administration of REGN1033 can also act to speed up muscle recovery and has the potential to alleviate muscle frailty in aged populations.

## Abbreviations

4832428D23Rik: methyltransferase like 21E
Actc1: actin, alpha, cardiac muscle 1
ActRIIB: activin Receptor IIB
ALK-4: activin-like kinase-4
ALK-5: activin-like kinase-5
CHO: Chinese hamster ovary
DAB: diaminobenzidine
Dkk3: dickkopf homologue 3
EDC: 1 ethyl-3-(3-dimethylaminopropyl) carbodiimide hydrochloride
EDTA: ethylenediaminetetraacetic acid
Fam65b: family with sequence similarity 65, member B
FCS: fetal calf serum
GA: gastrocnemius
GASP-1: growth and differentiation factor-associated serum protein-1
GDF11: growth and differentiation factor 11
HBS-T: HEPES-buffered Steinberg’s solution
HCL: hydrochloric acid
HEPES: 4-(2-hydroxyethyl)-1-piperazineethanesulfonic acid
HLS: hindlimb suspension
Igfn1: immunoglobulin-like and fibronectin type III domain containing 1
Mybph: myosin binding protein H
NHS: *N*-hydroxysuccinimide
PVDF: polyvinylidene fluoride
REGN1033: fully human monoclonal antibody against myostatin
SCID: severe combined immunodeficiency
SMAD: protein of SMA/MAD homology
TA: tibialis anterior
TGFβ: transforming growth factor beta
Tph1: tryptophan hydroxylase 1
Zmynd17: MSS51 mitochondrial translational activator
